# Health behaviors and life satisfaction among Polish classically trained singers: a cross-sectional study

**DOI:** 10.3389/fpubh.2026.1935698

**Published:** 2026-09-03

**Authors:** Cezary Roman, Mateusz Cybulski, Anna Zalewska, Bożena Czarkowska-Pączek, Anna Marchewka, Krystyna Rożek-Piechura

**Affiliations:** 1 Department of Clinical Phonoaudiology and Speech Therapy, Faculty of Health Sciences, Medical University of Bialystok, Bialystok, Poland; 2 Department of Integrated Medical Care, Faculty of Health Sciences, Medical University of Bialystok, Bialystok, Poland; 3 Department of Physiotherapy, Faculty of Health Sciences, University of Lomza, Lomza, Poland; 4 Department of Clinical Nursing, Medical University of Warsaw, Warsaw, Poland; 5 Department of Physiotherapy, University of Physical Education, Krakow, Poland; 6 Department of Physiotherapy in Internal Medicine and Oncology, University of Health and Sport Sciences, Wroclaw, Poland

**Keywords:** classically trained singers, health behaviors, health promotion, life satisfaction, occupational health, opera singers, performing-arts workforce, workforce health

## Abstract

**Introduction:**

Health behaviors and life satisfaction are important health-related outcomes in professional voice users, yet evidence among classically trained singers remains limited. This online cross-sectional study assessed health behaviors and life satisfaction among Polish classically trained singers and examined their associations with selected sociodemographic and occupational factors.

**Methods:**

Data were collected from May to July 2024 among 241 adult Polish classically trained singers, including professional singers and vocal students (151 women and 90 men; age range, 20–73 years). Health behaviors were assessed using the Health Behavior Inventory (HBI), and life satisfaction was assessed using the Satisfaction With Life Scale (SWLS). Group differences were examined using the Mann–Whitney U and Kruskal–Wallis tests, associations using Spearman's rank correlation coefficient, and multivariable linear regression models were fitted for the SWLS and total HBI scores with adjustment for age, sex, and type of vocal work. The Holm procedure was applied to account for multiple comparisons.

**Results:**

An average level of health behaviors was most common (42.7%), followed by low (32.4%) and high (24.9%) levels, and 55.2% of participants were classified in one of the satisfied-with-life categories. Life satisfaction was positively associated with the total HBI score (ρ = 0.508, 95% CI: 0.406–0.599; Holm-adjusted *p* < 0.001) and all four HBI domains (all adjusted *p* < 0.001), with the strongest association observed for positive mental attitude (ρ = 0.528, 95% CI: 0.429–0.616). After correction for multiple comparisons, women had higher scores than men for proper eating habits, health practices, and the total HBI score. In the multivariable analysis, sex was associated with the total HBI score, with women scoring 6.26 points higher than men after adjustment for age and type of vocal work (B = 6.257, 95% CI: 3.270–9.245; *p* < 0.001). No statistically significant adjusted associations with SWLS were observed for age, sex, or type of vocal work.

**Discussion:**

Health behaviors were positively associated with life satisfaction in this sample of Polish classically trained singers. The findings provide baseline evidence on health behaviors and life satisfaction in this specific performing-arts population and may inform further health-promotion research; however, the cross-sectional design precludes conclusions regarding temporal or causal relationships.

## 1 Introduction

Opera singers and other classically trained singers constitute a specific performing-arts workforce whose occupational activity depends on sustained vocal, physical, and psychological capacity. The voice is a central professional asset, and professional voice users therefore need to attend not only to vocal and orofacial function but also to broader aspects of health and wellbeing (1). Classical singing requires harmonically rich vocal timbre, precise articulatory control, and effective sound projection, typically developed through years of intensive training (2).

Classically trained singers are an occupationally relevant population because their professional and educational activities involve specific vocal, physical, and psychological demands. Previous research has highlighted the importance of considering repertoire, occupational workload, vocal habits, artistic goals, and health-related behaviors when examining the health needs of professional voice users (3, 4). In the present study, health behaviors and life satisfaction were assessed as distinct health-related outcomes.

Health behaviors comprise actions and established habits that may promote or undermine health. Behaviors relevant to both general and vocal health include avoiding tobacco use, limiting alcohol consumption, maintaining appropriate hydration and nutrition, engaging in physical activity, and applying preventive practices (5–8). These behaviors are particularly relevant to singers because health status, recovery capacity, and psychophysical fitness may be related to the ability to meet occupational demands.

Life satisfaction is a global cognitive evaluation of one's life as a whole, based on individually selected criteria. It is an important indicator of subjective wellbeing and may be associated with perceived health, interpersonal relationships, occupational circumstances, and work-life balance. Previous research has reported associations between life satisfaction and self-rated health, and studies involving musicians indicate that artistic activity may coexist with both meaningful professional engagement and health-related burdens (9–12).

Like other performing artists, opera singers may face irregular schedules, performance pressure, financial uncertainty, travel, and substantial responsibility for maintaining vocal and physical fitness. Occupational pressure may also lead some professional singers, particularly freelancers, to perform despite physical or mental health problems, which may be related to adverse consequences for health, occupational functioning, and overall wellbeing (13, 14). In this context, health behaviors and life satisfaction are relevant public health and occupational health outcomes rather than solely individual lifestyle characteristics.

Pain while singing, vocal fatigue, or a temporary inability to perform may be a major source of stress for singers, whose vocal ability is closely linked to professional identity, self-esteem, and their overall evaluation of life (13–17). Research on professional musicians indicates that occupational demands may coexist with physical and mental health problems, and that coping with work-related stress may be associated with wellbeing and life satisfaction (18, 19). Health-promoting behaviors have also been associated with more favorable perceptions of health, fewer health complaints, and better wellbeing (20, 21).

To our knowledge, this is the first study to assess both health behaviors and life satisfaction among Polish classically trained singers and to examine the association between these two outcomes in this population. The present findings therefore extend the available evidence on health and functioning among classically trained singers beyond specific clinical symptoms. Our previous publication based on the same broader survey sample focused on self-reported TMD symptoms and selected aspects of TMD-related knowledge (22), whereas the present study addresses broader behavioral and subjective wellbeing outcomes. These findings provide complementary information relevant to occupational health and health promotion, particularly because health behaviors and life satisfaction may represent potentially addressable areas for future wellness-oriented research, although the present cross-sectional design does not allow conclusions regarding causality or intervention effectiveness.

Baseline evidence on health behaviors and life satisfaction may help identify areas for future wellness-oriented education, preventive strategies, and health-promotion planning in the performing-arts workforce, complementing evidence from previous research on specific health conditions in this population.

The primary aim of the present study was therefore to assess health behaviors and life satisfaction among Polish classically trained singers. The secondary objectives were to examine the association between health behaviors and life satisfaction and to analyze both outcomes according to sex, age, type of vocal work, and performed repertoire.

The following research questions were formulated: (1) What are the levels of health behaviors and life satisfaction among Polish classically trained singers? (2) Are health behaviors associated with life satisfaction in the study sample? (3) Do health behaviors and life satisfaction differ according to sex, type of vocal work, and performed repertoire, and are these outcomes associated with participant age?

## 2 Materials and methods

### 2.1 Participants and procedure

This article reports findings on health behaviors and life satisfaction collected as part of a broader online cross-sectional survey of Polish classically trained singers. Findings on self-reported temporomandibular disorder (TMD) symptoms and TMD-related knowledge obtained from the same participant sample have been published separately. The present manuscript addresses distinct outcomes related to health behaviors and life satisfaction and does not report the descriptive TMD outcomes from the previous publication.

Data were collected between May and July 2024. The study included 241 Polish classically trained singers (151 women and 90 men) aged 20–73 years, representing different stages of formal vocal training and professional activity. The sample comprised professional opera singers and students enrolled in vocal programs at Polish music academies. Students were eligible because their formal education included training in classical operatic vocal technique.

Eligibility criteria included Polish citizenship, age ≥18 years, provision of informed consent, and either professional use of classical operatic singing technique or enrollment in a vocal program providing formal training in this technique. Professional participants included soloists, choir singers, chamber ensemble artists, secondary-school singing teachers, and academic singing teachers. Incorrectly completed questionnaires and responses from individuals with medical education were excluded. The exclusion of participants enrolled in medical education was an eligibility criterion of the broader survey because that survey also assessed TMD-related knowledge and was intended to reduce the potential influence of formal biomedical education on responses to the knowledge-related items. This criterion was therefore retained for the present analysis to preserve the same study sample; it was not introduced specifically in relation to the health behavior or life satisfaction outcomes examined in the present manuscript.

The survey was administered using Google Forms. Invitations containing study information, assurances of anonymity and voluntary participation, and an informed-consent statement were shared through the researchers' social media profiles and sent to opera houses, philharmonic institutions, musical theaters, and music universities in Poland. Because recruitment relied on open online and institutional distribution, neither the number of eligible individuals reached nor the response rate could be determined. No complete national sampling frame was available; consequently, recruitment was non-probability based. Participants could withdraw from the survey at any time without consequences. The study dataset is publicly available in the Open Research Data Repository (RepOD) (23).

The study protocol was approved by the Senate Committee for Research Ethics of the University of Lomza (approval No. 6508100; 23 April 2024). Before providing consent, all participants received information about the study procedures and applicable data-protection requirements. Personal data were processed in accordance with Regulation (EU) 2016/679 and the Polish Act of 10 May 2018 on the Protection of Personal Data.

### 2.2 Measures

The survey comprised three components: a sociodemographic and occupational questionnaire, the Polish adaptation of the Satisfaction With Life Scale (SWLS) by Zygfryd Juczyński (24), and the Polish adaptation of the Health Behavior Inventory (HBI) by Zygfryd Juczyński (25).

#### 2.2.1 Sociodemographic and occupational questionnaire

This section collected sociodemographic data and information on type of vocal work, performed repertoire, frequency of independent voice practice, and rehearsal frequency.

#### 2.2.2 Satisfaction with life Scale

The SWLS is a standardized five-item instrument. Participants rate their agreement with each statement on a seven-point scale ranging from 1 (“strongly disagree”) to 7 (“strongly agree”). Item scores are summed to yield a total score reflecting life satisfaction. Total scores range from 5 to 35, with lower scores indicating lower life satisfaction. Scores are interpreted as follows: 5–9 points indicate that the respondent is extremely dissatisfied with life; 10–14 points indicate that the respondent is dissatisfied with life; 15–19 points indicate that the respondent is slightly dissatisfied with life; 20 points indicate that the respondent is neither satisfied nor dissatisfied with life; 21–25 points indicate that the respondent is slightly satisfied with life; 26–30 points indicate that the respondent is satisfied with life; and 31–35 points indicate that the respondent is extremely satisfied with life (24). Reported Cronbach's α for the SWLS in adults exceeds 0.70 (24). In the present sample, Cronbach's α was 0.905.

#### 2.2.3 Health Behavior Inventory

The HBI is a standardized instrument comprising 24 health-related statements grouped into four domains: health practices (habits related to sleep, physical activity, and recreation), proper eating habits (types of food consumed), preventive behaviors (adherence to medical recommendations and active information seeking about illness and health), and positive mental attitude (avoidance of stress, depressing situations, tension, and excessively strong emotions). Each item is rated on a five-point Likert scale. Item scores are summed to obtain a total health behavior score, which is then converted to a standardized sten score. Sten scores of 1–4 indicate a low level of health behaviors, scores of 5–6 indicate an average level, and scores of 7–10 indicate a high level. Reported Cronbach's α for the HBI in adults exceeds 0.70 (25). In the present sample, Cronbach's α was 0.908 for the total HBI score and 0.811, 0.662, 0.773, and 0.701 for proper eating habits, preventive behaviors, positive mental attitude, and health practices, respectively.

### 2.3 Statistical analysis

Statistical analyses were performed using Statistica version 13 (TIBCO Software Inc., Palo Alto, CA, USA, 2017). Categorical variables were presented as frequencies and percentages, whereas numerical variables were summarized using means, standard deviations, medians, quartiles, and ranges. The Mann-Whitney U test was used to compare two independent groups, and the Kruskal-Wallis test was used for comparisons involving more than two groups. Spearman's rank correlation coefficient was used to assess associations between SWLS and HBI scores and between age and each of these measures.

For analyses by type of vocal work, chamber ensemble artists, secondary-school singing teachers, and academic singing teachers were combined into one category because of the small group sizes. Performed repertoire was analyzed inferentially in three categories: cantata-oratorio, song, and opera. Participants who selected “other” repertoire (*n* = 10) were retained in the descriptive repertoire table but were not included in inferential analyses by repertoire because this category was small and heterogeneous.

To account for multiple testing, the Holm procedure was applied separately within each prespecified family of analyses, including comparisons by sex, age group, type of vocal work, and performed repertoire, as well as the corresponding families of correlation analyses. For significant Kruskal-Wallis tests, pairwise comparisons were performed using Dunn's *post hoc* test with Holm adjustment. Effect sizes were calculated for non-parametric group comparisons: rank-biserial correlation (r_rb) for Mann-Whitney U tests and epsilon squared (ε^2^) for Kruskal-Wallis tests. Spearman's ρ was used as the measure of association for correlation analyses. Ninety-five percent confidence intervals (95% CIs) were reported for effect estimates where applicable.

To examine whether the two primary outcomes were associated with age, sex, and type of vocal work after mutual adjustment for these participant characteristics, two multivariable linear regression models were fitted with the SWLS total score and the HBI total score as dependent variables. Age was entered as a continuous variable, whereas sex and type of vocal work were entered as categorical variables. Men and vocal students served as the reference categories. The grouping of vocal work was the same as in the non-parametric analyses. Heteroskedasticity-consistent HC3 standard errors were used, and unstandardized regression coefficients (B) with 95% CIs and *p*-values were reported. Model fit was summarized using *R*^2^ and adjusted *R*^2^.

Internal consistency of the SWLS, the total HBI, and the four HBI domains in the present sample was assessed using Cronbach's α. All tests were two-sided, and statistical significance was set at *p* < 0.05.

## 3 Results

### 3.1 General characteristics

The sample comprised 151 women and 90 men aged 20–73 years. The mean age was 35.1 ± 11.3 years, and the median age was 33 years. Opera was the most commonly reported repertoire (60.2%), followed by song (19.9%), cantata-oratorio (15.8%), and other repertoire (4.1%). Participants most commonly reported practicing independently three times per week, while rehearsals were most commonly reported twice per week (Table 1).

**Table 1:** Characteristics of the study sample.

Characteristic	Category/statistic	Value
Age (years)	M	35.1
	Me	33
	SD	11.3
	Q1	24
	Q3	43
	Min.	20
	Max.	73
Performed repertoire	Cantata-oratorio	38 (15.8%)
	Song	48 (19.9%)
	Opera	145 (60.2%)
	Other	10 (4.1%)
Frequency of independent voice practice	Daily	17 (7.1%)
	6 times per week	8 (3.3%)
	5 times per week	27 (11.2%)
	4 times per week	42 (17.4%)
	3 times per week	64 (26.6%)
	2 times per week	48 (19.9%)
	Once per week	26 (10.8%)
	Less often	9 (3.7%)
Rehearsal frequency	Daily	3 (1.2%)
	6 times per week	5 (2.1%)
	5 times per week	14 (5.8%)
	4 times per week	15 (6.2%)
	3 times per week	35 (14.5%)
	2 times per week	83 (34.4%)
	Once per week	40 (16.6%)
	Less often	46 (19.1%)

M, mean; Me, median; SD, standard deviation; Q1, lower quartile; Q3, upper quartile; Min., minimum; Max., maximum.

### 3.2 Health behaviors and life satisfaction

Table 2 summarizes the four HBI domain scores, the total HBI score, and the classification of health behavior levels. Mean domain scores ranged from 3.31 for health practices to 3.49 for proper eating habits. An average level of health behaviors was most common (42.7%), followed by a low level (32.4%) and a high level (24.9%).

**Table 2:** Health behavior scores and HBI classification in the study sample.

Health behavior measure	M	Me	SD	Q1	Q3	Min.	Max.
Health behavior scores							
Proper eating habits	3.49	3.50	0.60	3.00	4.00	1.67	5.00
Preventive behaviors	3.32	3.33	0.56	3.00	3.67	1.67	5.00
Positive mental attitude	3.39	3.33	0.59	3.00	3.83	1.83	5.00
Health practices	3.31	3.33	0.56	3.00	3.67	1.67	5.00
Total HBI score	81.1	81	11.9	72	90	51	119
HBI classification							
Level of health behaviors	*n*			%			
Low	78			32.4%			
Average	103			42.7%			
High	60			24.9%			

HBI, Health Behavior Inventory; M, mean; Me, median; SD, standard deviation; Q1, lower quartile; Q3, upper quartile; Min., minimum; Max., maximum.

The mean SWLS score was 21.7 (SD = 4.9), with a median of 21. Overall, 59 participants (24.5%) were classified in one of the dissatisfied-with-life categories, 49 (20.3%) were classified as neither satisfied nor dissatisfied, and 133 (55.2%) were classified in one of the satisfied-with-life categories. The most common category was slightly satisfied (37.8%), whereas 2.5% were extremely satisfied (Table 3).

**Table 3:** Life satisfaction scores and classification in the study sample.

Life satisfaction measure	M	Me	SD	Q1	Q3	Min.	Max.
Life satisfaction scores							
SWLS	21.7	21	4.9	20	25	5	35
SWLS classification							
Life satisfaction classification	*n*			%			
Extremely dissatisfied	3			1.2%			
Dissatisfied	8			3.3%			
Slightly dissatisfied	48			19.9%			
Neither satisfied nor dissatisfied	49			20.3%			
Slightly satisfied	91			37.8%			
Satisfied	36			14.9%			
Extremely satisfied	6			2.5%			

SWLS, Satisfaction With Life Scale; M, mean; Me, median; SD, standard deviation; Q1, lower quartile; Q3, upper quartile; Min., minimum; Max., maximum.

Spearman's rank correlation analysis showed a positive association between life satisfaction and the total HBI score (ρ = 0.508, 95% CI: 0.406–0.599; Holm-adjusted *p* < 0.001). Life satisfaction was also positively associated with proper eating habits (ρ = 0.425, 95% CI: 0.314–0.525; adjusted *p* < 0.001), preventive behaviors (ρ = 0.425, 95% CI: 0.315–0.526; adjusted *p* < 0.001), positive mental attitude (ρ = 0.528, 95% CI: 0.429–0.616; adjusted *p* < 0.001), and health practices (ρ = 0.389, 95% CI: 0.272–0.497; adjusted *p* < 0.001). The strongest association was observed for positive mental attitude (Table 4).

**Table 4:** Correlations between SWLS and HBI scores.

HBI measure	Spearman's ρ	95% CI	*p*	Holm-adjusted *p*
Proper eating habits	0.425	0.314 to 0.525	<0.001	<0.001
Preventive behaviors	0.425	0.315 to 0.526	<0.001	<0.001
Positive mental attitude	0.528	0.429 to 0.616	<0.001	<0.001
Health practices	0.389	0.272 to 0.497	<0.001	<0.001
Total HBI score	0.508	0.406 to 0.599	<0.001	<0.001

HBI, Health Behavior Inventory; SWLS, Satisfaction With Life Scale; CI, confidence interval; ρ, Spearman's rank correlation coefficient. Holm-adjusted *p*-values account for multiple comparisons within this family of five correlations. The 95% CIs for Spearman's ρ were estimated using percentile bootstrap resampling.

### 3.3 Associations of selected factors with health behaviors and life satisfaction

After Holm correction for multiple comparisons, women had significantly higher scores than men for proper eating habits (adjusted *p* < 0.001), health practices (adjusted *p* = 0.022), and the total HBI score (adjusted *p* = 0.006). Differences in preventive behaviors and positive mental attitude did not remain statistically significant after correction (both adjusted *p* = 0.0504). SWLS scores did not differ between women and men (adjusted *p* = 0.961) (Table 5).

**Table 5:** SWLS and HBI scores according to sex.

Measure	Women (*n* = 151) M ±SD	Men (*n* = 90) M ±SD	U	r_rb (95% CI)	*p*	Holm-adjusted *p*
SWLS	21.7 ± 4.9	21.7 ± 5.0	6,815.0	0.004 (−0.151 to 0.155)	0.9609	0.9609
Proper eating habits	3.6 ± 0.6	3.3 ± 0.6	8,936.5	0.314 (0.172 to 0.452)	<0.001	<0.001
Preventive behaviors	3.4 ± 0.6	3.2 ± 0.6	8,043.0	0.183 (0.040 to 0.324)	0.0168	0.0504
Positive mental attitude	3.5 ± 0.6	3.3 ± 0.6	8,027.0	0.181 (0.034 to 0.326)	0.0180	0.0504
Health practices	3.4 ± 0.6	3.2 ± 0.6	8,262.0	0.213 (0.062 to 0.359)	0.0054	0.0216
Total HBI score	83.5 ± 11.6	77.2 ± 11.6	8,508.0	0.249 (0.101 to 0.391)	0.0012	0.0060

HBI, Health Behavior Inventory; SWLS, Satisfaction With Life Scale; M, mean; SD, standard deviation; r_rb, rank-biserial correlation; CI, confidence interval. Mann–Whitney U tests. Positive rank-biserial correlations indicate higher scores among women. Holm-adjusted *p*-values account for multiple comparisons within this family of six tests.

No statistically significant differences across age groups remained after correction for multiple comparisons. Although the unadjusted Kruskal–Wallis test for SWLS yielded *p* = 0.0191, this difference was not statistically significant after Holm correction (adjusted *p* = 0.1146; ε^2^ = 0.029). No statistically significant differences were observed for the total HBI score or any HBI domain (Table 6).

**Table 6:** SWLS and HBI scores according to age group.

Measure	<25 years (*n* = 61), M ±SD	25–34 years (*n* = 72), M ±SD	35–44 years (*n* = 59), M ±SD	≥45 years (*n* = 49), M ±SD	*p*	ε^2^	Holm-adjusted *p*
SWLS	21.0 ± 4.0	20.8 ± 5.4	22.2 ± 5.2	23.3 ± 4.7	0.0191	0.029	0.1146
HBI–proper eating habits	3.42 ± 0.60	3.44 ± 0.60	3.59 ± 0.60	3.52 ± 0.58	0.2958	0.003	1.000
HBI–preventive behaviors	3.27 ± 0.50	3.30 ± 0.54	3.38 ± 0.61	3.36 ± 0.62	0.4603	<0.001	1.000
HBI–positive mental attitude	3.33 ± 0.61	3.35 ± 0.57	3.46 ± 0.59	3.45 ± 0.61	0.4378	<0.001	1.000
HBI–health practices	3.30 ± 0.61	3.24 ± 0.49	3.34 ± 0.59	3.39 ± 0.56	0.3214	0.002	1.000
HBI–total score	79.9 ± 12.5	80.0 ± 11.4	82.6 ± 11.7	82.3 ± 12.2	0.3479	0.001	1.000

HBI, Health Behavior Inventory; SWLS, Satisfaction With Life Scale; M, mean; SD, standard deviation; ε^2^, epsilon squared. Kruskal–Wallis tests. Holm-adjusted *p*-values account for multiple comparisons within this family of six tests.

In the total sample, age showed a weak positive correlation with SWLS (ρ = 0.188, 95% CI: 0.068–0.305; Holm-adjusted *p* = 0.020). This association was also observed among men (ρ = 0.292, 95% CI: 0.104–0.469; adjusted *p* = 0.031), but not among women (ρ = 0.129, 95% CI: −0.023–0.278; adjusted *p* = 0.687). None of the correlations between age and the total HBI score or individual HBI domains remained statistically significant after correction for multiple comparisons (Table 7).

**Table 7:** Correlations of age with SWLS and HBI scores, overall and by sex.

Measure	Overall (*n* = 241), ρ	95% CI	*p*	Holm-adjusted *p*	Women (*n* = 151), ρ	95% CI	*p*	Holm-adjusted *p*	Men (*n* = 90), ρ	95% CI	*p*	Holm-adjusted *p*
SWLS	**0.188**	0.068 to 0.305	0.0034	**0.0203**	0.129	−0.023 to 0.278	0.1144	0.6866	**0.292**	0.104 to 0.469	0.0052	**0.0314**
Proper eating habits	0.088	−0.037 to 0.210	0.1723	0.7805	0.100	−0.062 to 0.254	0.2237	1.000	0.153	−0.055 to 0.355	0.1494	0.1494
Preventive behaviors	0.066	−0.059 to 0.194	0.3061	0.7805	0.004	−0.162 to 0.173	0.9633	1.000	0.233	0.034 to 0.422	0.0269	0.0985
Positive mental attitude	0.092	−0.038 to 0.218	0.1561	0.7805	0.046	−0.116 to 0.215	0.5746	1.000	0.212	0.002 to 0.408	0.0452	0.0985
Health practices	0.088	−0.036 to 0.215	0.1722	0.7805	0.012	−0.149 to 0.175	0.8801	1.000	0.261	0.057 to 0.452	0.0130	0.0651
Total HBI score	0.091	−0.039 to 0.223	0.1573	0.7805	0.057	−0.111 to 0.222	0.4888	1.000	0.237	0.024 to 0.436	0.0246	0.0985

HBI, Health Behavior Inventory; SWLS, Satisfaction With Life Scale; values are Spearman's rank correlation coefficients (ρ). Holm-adjusted *p*-values account for multiple comparisons within each set of six correlations. The 95% CIs for Spearman's ρ were estimated using percentile bootstrap resampling. Bold values identify results that remained statistically significant after Holm adjustment (Holm-adjusted *p* < 0.05).

After correction for multiple comparisons, SWLS scores differed significantly according to type of vocal work (Holm-adjusted *p* = 0.029; ε^2^ = 0.042). Dunn's *post hoc* tests with Holm adjustment indicated higher SWLS scores among soloists than students (adjusted *p* = 0.035) and among participants in other professional roles than students (adjusted *p* = 0.035). No other pairwise comparisons were statistically significant. Although the unadjusted Kruskal-Wallis tests indicated differences in preventive behaviors (*p* = 0.0346) and positive mental attitude (*p* = 0.0174), these findings did not remain statistically significant after Holm correction (adjusted *p* = 0.138 and *p* = 0.087, respectively). No statistically significant differences were observed for proper eating habits, health practices, or the total HBI score after correction (Table 8).

**Table 8:** SWLS and HBI scores according to type of vocal work.

Measure	Student (*n* = 64), M ±SD	Choir Singer (*n* = 62), M ±SD	Soloist (*n* = 63), M ±SD	Other roles (*n* = 52), M ±SD	ε^2^	*p*	Holm-adjusted *p*
SWLS	20.77 ± 4.27	20.84 ± 4.39	22.65 ± 6.14	22.63 ± 4.41	0.042	0.0049	**0.0293**
HBI–proper eating habits	3.45 ± 0.63	3.39 ± 0.53	3.61 ± 0.65	3.53 ± 0.56	0.012	0.1167	0.2335
HBI–preventive behaviors	3.31 ± 0.52	3.22 ± 0.64	3.47 ± 0.57	3.29 ± 0.49	0.024	0.0346	0.1382
HBI–positive mental attitude	3.38 ± 0.58	3.27 ± 0.63	3.56 ± 0.62	3.34 ± 0.48	0.030	0.0174	0.0872
HBI–health practices	3.31 ± 0.61	3.24 ± 0.56	3.37 ± 0.58	3.32 ± 0.47	0.000	0.6235	0.6235
HBI–total score	80.67 ± 12.57	78.69 ± 12.35	84.08 ± 11.44	80.87 ± 10.59	0.019	0.0604	0.1811

HBI, Health Behavior Inventory; SWLS, Satisfaction With Life Scale; M, mean; SD, standard deviation. ε^2^, epsilon squared. Kruskal–Wallis tests were used for comparisons according to type of vocal work. Holm-adjusted *p*-values account for multiple comparisons within this family of six tests. The “other roles” category comprised chamber ensemble artists (*n* = 10), secondary-school singing teachers (*n* = 24), and academic singing teachers (*n* = 18). Dunn's *post hoc* tests with Holm adjustment showed higher SWLS scores among soloists than students (adjusted *p* = 0.035) and among participants in other professional roles than students (adjusted *p* = 0.035). No other pairwise comparisons were statistically significant after adjustment. Bold values identify results that remained statistically significant after Holm adjustment (Holm-adjusted *p* < 0.05).

No statistically significant differences in SWLS, the total HBI score, or any HBI domain were observed according to performed repertoire, either before or after correction for multiple comparisons (all Holm-adjusted *p* > 0.05; Table 9). Effect sizes were negligible to very small (ε^2^ = 0.000–0.007).

**Table 9:** SWLS and HBI scores according to performed repertoire.

Measure	Cantata-oratorio (*n* = 38), M ±SD	Song (*n* = 48), M ±SD	Opera (*n* = 145), M ±SD	ε^2^	*p*	Holm-adjusted *p*
SWLS	22.08 ± 4.28	21.98 ± 5.40	21.62 ± 4.84	0.000	0.6932	1.000
HBI–proper eating habits	3.50 ± 0.59	3.48 ± 0.58	3.50 ± 0.60	0.000	0.8913	1.000
HBI–preventive behaviors	3.42 ± 0.57	3.26 ± 0.55	3.34 ± 0.56	<0.001	0.3484	1.000
HBI–positive mental attitude	3.47 ± 0.57	3.25 ± 0.65	3.43 ± 0.58	0.007	0.1631	0.9784
HBI–health practices	3.43 ± 0.49	3.25 ± 0.50	3.32 ± 0.58	0.003	0.2602	1.000
HBI–total score	82.92 ± 12.01	79.46 ± 11.56	81.45 ± 11.99	0.000	0.4279	1.000

HBI, Health Behavior Inventory; SWLS, Satisfaction With Life Scale; M, mean; SD, standard deviation. ε^2^, epsilon squared. Kruskal–Wallis tests were used for comparisons according to performed repertoire. Holm-adjusted *p*-values account for multiple comparisons within this family of six tests. Participants reporting “other” repertoire (*n* = 10) were retained in the descriptive characteristics but excluded from inferential analyses by repertoire because of the small and heterogeneous nature of this category.

### 3.4 Multivariable analyses

In multivariable analyses including age, sex, and type of vocal work simultaneously, sex was associated with the total HBI score. Women had, on average, a 6.26-point higher total HBI score than men after adjustment for age and type of vocal work (*B* = 6.257, 95% CI: 3.270–9.245; *p* < 0.001). Age and type of vocal work were not statistically significantly associated with the total HBI score in the adjusted model.

For SWLS, none of the included variables was statistically significant after mutual adjustment. In particular, the differences in SWLS observed across types of vocal work in the univariable analysis were not statistically significant after adjustment for age and sex. The SWLS model explained 4.9% of the variance (adjusted *R*^2^ = 0.029), whereas the total HBI model explained 9.7% (adjusted *R*^2^ = 0.078) (Table 10).

**Table 10:** Multivariable linear regression models for SWLS and total HBI scores.

Variable	SWLS B (95% CI)	*p*	Total HBI B (95% CI)	*p*
Age, per year	0.065 (−0.014 to 0.144)	0.105	0.147 (−0.045 to 0.338)	0.133
Women vs. men	0.612 (−0.686 to 1.911)	0.355	**6.257 (3.270 to 9.245)**	**<0.001**
Choir singer vs. student	−0.911 (−2.921 to 1.099)	0.374	−4.378 (−9.379 to 0.623)	0.086
Other roles vs. student	0.657 (−1.620 to 2.935)	0.572	−2.137 (−7.629 to 3.355)	0.446
Soloist vs. student	0.784 (−1.577 to 3.145)	0.515	1.027 (−3.919 to 5.973)	0.684

B, unstandardized regression coefficient; CI, confidence interval; HBI, Health Behavior Inventory; SWLS, Satisfaction With Life Scale. Multivariable linear regression models included age, sex, and type of vocal work simultaneously. Men and vocal students served as the reference categories. Other roles included chamber ensemble artists, secondary-school singing teachers, and academic singing teachers. Heteroskedasticity-consistent HC3 standard errors were used. Model fit: SWLS model, *R*^2^ = 0.049, adjusted *R*^2^ = 0.029; total HBI model, *R*^2^ = 0.097, adjusted *R*^2^ = 0.078. Bold values indicate statistically significant results (*p* < 0.05).

## 4 Discussion

The present study examined health behaviors and life satisfaction among Polish classically trained singers from an occupational health and public health perspective. Overall, the findings indicate variability in both health behaviors and life satisfaction within this performing-arts workforce. Several differences were observed in the univariable analyses; however, their interpretation should take into account the results of the multivariable models. After simultaneous adjustment for age, sex, and type of vocal work, sex remained associated with the total Health Behavior Inventory score, whereas none of these variables was statistically significantly associated with life satisfaction measured using the Satisfaction With Life Scale. These findings provide a more nuanced description of health behaviors and life satisfaction among the surveyed singers and may help identify questions relevant to future occupational health and health-promotion research.

In recent years, increasing attention has been given to comprehensive approaches to singers' health that encompass both somatic and psychosocial aspects (26). Appropriate vocal habits, vocal hygiene, and everyday health-promoting behaviors may be especially important for individuals whose voice is their primary occupational tool. Prestes et al. (27) reported unfavorable vocal-health behaviors among church singers, including tobacco use and excessive alcohol consumption. Ravall and Simberg (28) found that choir singers reported some behaviors potentially harmful to the voice more frequently than non-singers.

Dassie-Leite et al. (29) described additional factors relevant to singers' health, including insufficient hydration, sleep problems, improper eating habits, exposure to loud or smoke-filled environments, and inappropriate vocal technique. Stress, financial instability, and irregular work schedules may also hinder engagement in health-promoting behaviors. These factors may be relevant not only to vocal function but also to the broader health and wellbeing of singers. In the present study, an average level of health behaviors was most common (42.7%). A low level was identified in 32.4% of participants, whereas 24.9% had a high level. These findings indicate substantial variation in health behavior levels within the study sample and may help identify questions for further health-promotion research. However, because the study lacked a control group, it cannot determine whether health behaviors in this sample were more or less favorable than those in the general population or other occupational groups.

After correction for multiple comparisons, women had higher scores than men for proper eating habits and health practices, as well as for the total HBI score. Differences in preventive behaviors and positive mental attitude did not remain statistically significant after correction. Importantly, sex remained associated with the total HBI score in the multivariable analysis, with women scoring approximately 6.3 points higher than men after adjustment for age and type of vocal work. This findings may be considered in the context of evidence that women more frequently report health problems, monitor their health, and use healthcare services (4). However, the present study did not assess healthcare use or individual motivations for engaging in health-promoting behaviors; these factors therefore cannot be regarded as direct explanations for the observed differences. The findings concerning age were less consistent. Age showed a weak positive correlation with SWLS in the total sample, and this association remained statistically significant after correction for multiple comparisons. However, differences in SWLS across categorical age groups did not remain statistically significant after Holm correction, and age was not statistically significantly associated with SWLS in the multivariable model after adjustment for sex and type of vocal work. Although the age–SWLS correlation also remained statistically significant among men in the sex-stratified analysis, this does not establish that the association differed between women and men because no formal interaction analysis was performed. None of the correlations between age and HBI scores remained statistically significant after correction for multiple comparisons. Previous research has suggested that relationships between age and health behaviors may vary according to sex and the specific health behaviors examined (30). Overall, these findings provide only limited evidence for an association between age and life satisfaction in this sample and do not indicate a clear age-related pattern.

Unadjusted analyses suggested differences in preventive behaviors and positive mental attitude according to type of vocal work; however, these differences did not remain statistically significant after correction for multiple comparisons. Accordingly, the descriptively higher mean scores observed among soloists should not be interpreted as evidence of differences between occupational groups. Previous research has indicated that occupational demands and working conditions may be relevant to health-related behaviors and wellbeing among performing artists (11). In the multivariable analysis in the present study, type of vocal work was not statistically significantly associated with the total HBI score after adjustment for age and sex. Overall, the present findings do not provide robust evidence of differences in health behaviors according to type of vocal work, and further research is needed to examine whether occupational characteristics are related to health behaviors in classically trained singers.

More broadly, group singing has been examined in relation to wellbeing and psychosocial outcomes among children and young people, although findings from these populations cannot be directly extrapolated to adult classically trained singers (31).

Health problems among individuals undergoing music education have also been described in the literature. Undergraduate and postgraduate students at 10 conservatories in the United Kingdom had less favorable outcomes than their peers in the general population in relation to health responsibility, stress management, sleep quality, self-rated health, and the use of coping strategies (32). This finding suggests that intensive artistic education may be accompanied by distinct health and psychosocial burdens. However, direct comparison with the present findings is limited by differences in sample characteristics, measurement instruments, and recruitment methods.

Although many classical musicians derive enjoyment and satisfaction from their work, they may simultaneously experience adverse consequences related to the demands of professional life (11). In a large US study, 76% of 2,212 orchestral musicians reported serious problems affecting their ability to work and function in everyday life (33). Musculoskeletal complaints were among the frequently reported difficulties. The literature also highlights mental health problems among professional musicians (34), including chronic stress, sleep disorders, and the use of psychotherapy and psychotropic medication (35, 36). In the present sample, 55.2% of participants were classified in one of the satisfied-with-life categories, 20.3% were classified as neither satisfied nor dissatisfied, and 24.5% were classified in one of the dissatisfied-with-life categories. The most common classification was “slightly satisfied,” whereas only 2.5% were classified as extremely satisfied with life. These findings describe life satisfaction in the study sample but do not establish whether it was lower or higher than that in the general population or other occupational groups.

Life satisfaction did not differ by sex, which is consistent with the cited findings for the Polish population reported by the Public Opinion Research Center (37). Age-related differences in life satisfaction have been discussed in previous studies in relation to personal and professional circumstances (38–40). In the present study, however, the findings concerning age were not consistent across the different analytical approaches and therefore do not support a clear age-related pattern in life satisfaction among classically trained singers.

Life satisfaction did not differ by sex, which is consistent with the cited findings for the Polish population reported by the Public Opinion Research Center (37). Previous studies have discussed age-related differences in life satisfaction in relation to personal and professional circumstances (38–40); however, the present findings provide only limited evidence of an association between age and life satisfaction among classically trained singers.

Type of vocal work was associated with SWLS in the univariable analysis after correction for multiple comparisons, with *post hoc* comparisons indicating higher SWLS scores among soloists and participants in other professional roles than among students. However, type of vocal work was not statistically significantly associated with SWLS after adjustment for age and sex in the multivariable model. Moreover, the differences observed in preventive behaviors and positive mental attitude in the initial group comparisons did not remain statistically significant after correction for multiple comparisons. Taken together, these findings do not provide robust evidence that type of vocal work itself was associated with life satisfaction or health behaviors in this sample. The differences observed in univariable comparisons should therefore be interpreted cautiously (41–43).

Performed repertoire was not associated with life satisfaction, the total HBI score, or any assessed HBI domain, and this conclusion was unchanged after correction for multiple comparisons. This finding suggests that repertoire type alone was insufficient to differentiate the analyzed outcomes. However, the study did not include detailed measures of singing hours, rehearsal intensity, repertoire difficulty, or total occupational workload.

A principal finding was the positive association between life satisfaction and health behaviors. SWLS was positively correlated with the total HBI score and all four HBI domains, and all of these associations remained statistically significant after correction for multiple comparisons. The strongest association was observed for positive mental attitude. The partial conceptual overlap between these constructs should be considered: positive mental attitude encompasses behaviors and beliefs related to psychological functioning, whereas the SWLS provides a subjective cognitive evaluation of one's life. The observed correlations do not establish the direction of the associations. More favorable health behaviors may coexist with greater life satisfaction, but individuals who are more satisfied with their lives may also have greater resources and motivation to care for their health. Both variables may additionally be related to other factors, including health status, financial circumstances, employment stability, social support, sleep quality, stress, and workload.

The present findings may help identify questions for further health-promotion research among classically trained singers rather than provide evidence for specific interventions. Future studies could examine health behaviors and life satisfaction alongside occupationally relevant factors such as workload, recovery, stress, and work-life balance, which were not directly assessed in the present study. Prospective and intervention studies would be required before recommendations regarding the effectiveness of specific occupational health or health-promotion strategies could be made (7, 18, 44–46).

### 4.1 Strengths and limitations

Strengths of this study include the use of standardized instruments to assess health behaviors and life satisfaction and the inclusion of a relatively diverse group of individuals at different stages of vocal education and professional careers. This diversity enabled the analyzed variables to be described among both students and individuals engaged in different forms of work related to classical singing. To our knowledge, this is the first study to assess both health behaviors and life satisfaction among Polish classically trained singers and to examine the association between these two outcomes in this population. The present findings add to the available evidence on health and functioning among classically trained singers beyond specific clinical symptoms. Our previous publication based on the same broader survey sample focused on self-reported TMD symptoms and selected aspects of TMD-related knowledge (22), whereas the present study addresses broader behavioral and subjective wellbeing outcomes. The present findings therefore provide complementary evidence relevant to occupational health and health-promotion research in this specific performing-arts population.

The cross-sectional design precludes conclusions regarding the temporal or causal direction of the observed associations. All information was self-reported and may have been affected by recall bias, individual interpretation of the questions, and social desirability. The use of self-report instruments for both primary constructs may also have increased the observed correlations through common method variance.

Participants were recruited voluntarily through online and institutional channels. This recruitment strategy may have introduced self-selection bias, as individuals with greater interest in health-related topics may have been more likely to participate. No complete sampling frame was available, and the response rate could not be determined. The sample should therefore not be considered representative of all Polish classically trained singers. The absence of a control group also limits comparisons with the general population and other occupational groups.

The sample included both students and professional singers representing different occupational roles, which increased its heterogeneity. Small numbers in some categories required their combination into broader groups, potentially limiting the identification of more specific differences.

The study did not assess several variables that may be associated with health behaviors and life satisfaction, including current health status, chronic conditions, financial circumstances, employment stability, stress, sleep quality, social support, hours spent working and singing, and total occupational workload. These factors should be considered in future research.

This study forms part of a broader research project evaluating different aspects of health and functioning among Polish classically trained singers. The same participant sample was used in a separate publication addressing self-reported TMD symptoms and selected aspects of TMD-related knowledge (22). The present manuscript addresses distinct outcomes–health behaviors and life satisfaction–and their associations with selected sociodemographic and occupational variables.

## 5 Conclusion

In this sample, an average level of health behaviors was most common, and more than half of participants were classified in one of the satisfied-with-life categories. Life satisfaction was positively associated with the total HBI score and all four HBI domains, with the strongest association observed for positive mental attitude. These associations remained statistically significant after correction for multiple comparisons. After correction for multiple comparisons, women had higher scores than men for proper eating habits, health practices, and the total HBI score. In the multivariable analysis, sex remained associated with the total HBI score after adjustment for age and type of vocal work. Although age was weakly positively correlated with life satisfaction, age was not statistically significantly associated with SWLS after adjustment for sex and type of vocal work. Similarly, differences in SWLS according to type of vocal work observed in univariable analyses were not statistically significant after adjustment for age and sex. Performed repertoire was not associated with the analyzed outcomes. These findings provide baseline evidence on health behaviors and life satisfaction in this sample of Polish classically trained singers and may inform further occupational health and health-promotion research in performing-arts populations. Prospective and intervention studies are needed before recommendations regarding specific occupational health or health-promotion strategies can be made.

## Data Availability

The datasets presented in this study can be found in online repositories. The names of the repository/repositories and accession number(s) can be found below: The data are openly available in the Open Research Data Repository (RepOD). Dataset DOI/accession number: https://doi.org/10.18150/39XSME.
